# Identification and Functional Characterisation of Novel Glucokinase Mutations Causing Maturity-Onset Diabetes of the Young in Slovakia

**DOI:** 10.1371/journal.pone.0034541

**Published:** 2012-04-06

**Authors:** Lucia Valentínová, Nicola L. Beer, Juraj Staník, Nicholas D. Tribble, Martijn van de Bunt, Miroslava Hučková, Amy Barrett, Iwar Klimeš, Daniela Gašperíková, Anna L. Gloyn

**Affiliations:** 1 Institute of Experimental Endocrinology, Slovak Academy of Science, Bratislava, Slovakia; 2 Oxford Centre for Diabetes Endocrinology and Metabolism, University of Oxford, Oxford, United Kingdom; 3 Children Diabetes Centre, First Department of Paediatrics, Comenius University, Bratislava, Slovakia; 4 Centre for Molecular Medicine, Slovak Academy of Sciences, Bratislava, Slovakia; 5 NIHR Oxford Biomedical Research Centre, Oxford, United Kingdom; University of California San Francisco, United States of America

## Abstract

Heterozygous glucokinase (*GCK*) mutations cause a subtype of maturity-onset diabetes of the young (GCK-MODY). Over 600 *GCK* mutations have been reported of which ∼65% are missense. In many cases co-segregation has not been established and despite the importance of functional studies in ascribing pathogenicity for missense variants these have only been performed for <10% of mutations. The aim of this study was to determine the minimum prevalence of GCK-MODY amongst diabetic subjects in Slovakia by sequencing *GCK* in 100 Slovakian probands with a phenotype consistent with GCK-MODY and to explore the pathogenicity of identified variants through family and functional studies.

Twenty-two mutations were identified in 36 families (17 missense) of which 7 (I110N, V200A, N204D, G258R, F419S, c.580-2A>C, c.1113–1114delGC) were novel. Parental DNA was available for 22 probands (covering 14/22 mutations) and co-segregation established in all cases. Bioinformatic analysis predicted all missense mutations to be damaging. Nine (I110N, V200A, N204D, G223S, G258R, F419S, V244G, L315H, I436N) mutations were functionally evaluated. Basic kinetic analysis explained pathogenicity for 7 mutants which showed reduced glucokinase activity with relative activity indices (RAI) between 0.6 to <0.001 compared to wild-type GCK (1.0). For the remaining 2 mutants additional molecular mechanisms were investigated. Differences in glucokinase regulatory protein (GKRP) –mediated-inhibition of GCK were observed for both L315H & I436N when compared to wild type (IC_50_ 14.6±0.1 mM & 20.3±1.6 mM vs.13.3±0.1 mM respectively [p<0.03]). Protein instability as assessed by thermal lability studies demonstrated that both L315H and I436N show marked thermal instability compared to wild-type GCK (RAI at 55°C 8.8±0.8% & 3.1±0.4% vs. 42.5±3.9% respectively [p<0.001]). The minimum prevalence of GCK-MODY amongst Slovakian patients with diabetes was 0.03%.

In conclusion, we have identified 22 *GCK* mutations in 36 Slovakian probands and demonstrate that combining family, bioinformatic and functional studies can aid the interpretation of variants identified by molecular diagnostic screening.

## Introduction

Heterozygous inactivating mutations in the glucokinase gene (*GCK*) are one cause of maturity-onset diabetes of the young (MODY) characterized by stable elevated fasting plasma glucose levels [1], [2]. Micro and macrovascular complications are rare and patients can usually be treated by diet modification alone [3]. A molecular diagnosis is important because there are implications for treatment, prognosis and family members [3]. The prevalence of GCK-MODY is difficult to assess as the mild hyperglycaemia and absence of symptoms mean that many patients are not diagnosed. Large scale population based studies have not been performed but a number of studies have ascertained the prevalence of GCK-MODY in subjects with impaired glucose tolerance and diabetes [4], . There are differences in prevalence across these studies which likely reflect both the age of the population tested and the ascertainment criteria [12], [13], [14].

Glucokinase is a key enzyme in glycolysis which has been termed the pancreatic glucose sensor on account of its kinetic properties which result in the threshold for glucose stimulated insulin release (GSIR) being around 5 mmol/L [15]. Heterozygous inactivating *GCK* mutations shift the set point for GSIR, resulting in elevated fasting plasma glucose levels (typically between 5.5–8.0 mmol/L in individuals with *GCK* mutations [16], [17]). The majority of individuals with GCK-MODY have a small (<3.5 mmol/L) 2 hour glucose increment following an oral glucose tolerance test (OGTT) which can help distinguish them from patients with other forms of MODY [18].

Over 600 *GCK* mutations have been reported and in a recent review by Osbak and colleagues over 65% (402/620) are missense, with 56% only been reported in a single family [12]. There is increasing recognition of the difficulties in ascribing pathogenicity to missense variants uncovered through medical resequencing projects. Despite the importance of establishing co-segregation this is often over looked and functional studies have only been performed for a minority (<10%) of missense mutations [12]. A recent study has highlighted the importance of using a combination of approaches to correctly assign pathogenicity for *GCK* missense mutations [19]. In this study, we report an investigation of the minimum prevalence of GCK-MODY in Slovakia followed by an exploration of the pathogenicity of identified variants through family, bioinformatic and functional studies.

## Materials and Methods

### Subjects

One hundred probands collected between years 2003–2010 underwent diagnostic screening for *GCK* mutations following referral from their diabetologist. Selection criteria for testing were persistent and stable fasting hyperglycemia (5.5–10.0 mmol/L), HbA1c<8% [64 mmol/ml] (DCCT scale), age of diagnosis <35 years, and where on insulin treatment, the dose was <0.4 IU/Kg/day. Informed written consent was obtained from all subjects. For subjects under the age of 18 years written consent was obtained from either a parent or legal guardian.

### Mutation Screening

Genomic DNA was extracted from peripheral blood using the Flexi Gene kit (Qiagen Ltd, Crawley, UK). The *GCK* neuroendocrine promoter, exons 1a and 2–10, and all intron-exon boundaries were amplified by PCR using previously published primers [20], and sequenced bi-directionally. Sequences were compared to the reference genomic *GCK* sequence (NM_000162.3) using SeqScape (version 2.1.1; Applied Biosystems). Partial or entire *GCK* deletions were excluded by multiplex ligation-dependent probe amplification (MLPA) using the P241-B MODY kit (MRC-Holland, Amsterdam, The Netherlands). Results were analyzed using GeneMarker version 1.95 (Soft Genetics, State College, PA, USA).

### Phenotypic characterisation

Plasma glucose concentrations were measured via the glucose oxidase method (Hitachi 911, Hitachinaka, Japan), whilst cholesterol, HDL-cholesterol and triglyceride levels were measured using standard enzymatic protocols (Diasys diagnostic systems, Holzheim, Germany; Randox Laboratories Ltd. Crumlin, UK).

### *In silico* structural analysis

All missense mutations were analysed *in silico* using SIFT, PolyPhen, and Mutation Taster (http://sift.jcvi.org/, http://genetics.bwh.harvard.edu/pph/ and http://www.mutationtaster.org/ respectively). Three-dimensional stuctural analysis was performed using PyMOL version 1.2 (DeLano Scientific, South San Francisco, CA, USA).

### Preparation of recombinant proteins

Two preparations of human wild-type and each GCK mutant were generated as recombinant gluthationyl S-transferase-tagged fusion proteins using previously-described protocols [21], [22], [23]. Recombinant human wild-type glucokinase regulatory protein (GKRP) was also prepared as previously published [24], [25].

### Enzyme kinetics

The kinetic properties of each GCK enzyme were determined using glucose 6-phosphate dehydrogenase-coupled assays [21], [22]. The relative activity index (RAI) for each enzyme and the predicted threshold for glucose stimulated insulin release (GSIR) were calculated as previously described [17]. Inhibition assays with human GKRP were performed in line with previous studies [24], [25], [26].

### Thermolability Assays

Thermal instability assays were carried-out using previously described protocols [22], [27], but with the following modifications. Each GCK enzyme was incubated over a 40–63°C temperature range for 30 minutes. Immediately after incubation, enzymes were diluted in an assay containing 100 mmol/L HEPES, 6 mmol/L MgCl_2_, 150 mmol/L KCl, 2 mmol/L DTT(prior to GCK activity measurement) [22], [28], [29].

### GCK-MODY frequency data

The minimum prevalence of GCK-MODY in Slovakia was calculated based on available data for the total number of diabetic patients, and the total number of inhabitants in Slovakia based on the Slovak National Health Information Center (NCZI, www.nczisk.sk).

### Statistical Analysis

Continuous variables are reported as mean plus or minus the standard error of the mean (SEM). Comparisons amongst variables were carried out using independent sample t-tests on SPSS software (version 17.0, IBM, Chicago, IL, USA). Incontinous variables are reported as median with a given range.

## Results

### *GCK* mutational screening and clinical characterisation

Mutational screening identified 22 *GCK* mutations in 36 of the probands (**Table 1**); 17 missense, 1 frame-shift, 2 in frame deletions, 1 promoter and 1 splice-site. Seven of these mutations were novel (I110N, V200A, N204D, G258R, F419S, c.580-2A>C, c.1113-1114delGC), they were also absent from 200 control chromosomes from normoglycaemic individuals. No variants were present in the October 2011 release of the 1000 Genomes project (http://www.1000genomes.org/). All novel mutations except G258R were identified in a single family, whilst G258R was identified in 2 unrelated families. Parental DNA was available for 22 probands (covering 14/22 mutations) and co-segregation was established in all cases (**Figure S1**). No partial or whole *GCK* deletions were identified. The clinical and biochemical characteristics of the *GCK* mutation carriers are presented in **table 2**. The median proband age is consistent with other studies which have evaluated diabetes subtype [30].

**Table 1:** *GCK* mutations identified in Slovakian probands with a phenotype of GCK-MODY.

Region	Abbreviated protein nomenclature	Nucleotide nomenclature	Number of probands with mutation	Reported functional studies	References
Islet promoter	-	c.−71G>C	9	Y	[42]
exon 2	R36W	c.106C>T	1	Y	[43]
exon 2	R43H	c.128G>A	1	N	[35]
exon 3	G72R	c.214G>A	1	Y	[44], [45]
exon 3	I110N	c.329T>A	1	-	Novel
exon 4	F150del	c.449–451delTCT	1	N	[8], [45]
exon 4	S151del	c.451–453delTCC	1	N	[44]
exon 5	A188T	c.562G>A	1	Y	[46]
intron 5	-	c.580 -2A>C	1	-	Novel
exon 6	V200A	c.599C>T	1	-	Novel
exon 6	N204D	c.610A>G	1	-	Novel
exon 6	T206P	c.616A>C	1	N	[12]
exon 6	G223S	c.667G>A	1	N	[8], [44]
exon 7	T228M	c.683C>T	1	N	[47]
exon 7	V244G	c.731T>G	2	N	[12]
exon 7	M251I	c.752T>G	1	N	[8]
exon 7	G258R	c.772G>C	2	-	Novel
exon 8	L315H	c.944T>A	2	N	[48]
exon 8	G318R	c.352G>A	3	N	[4]
exon 9	A370fs	c.1113–1114delCG	1	-	Novel
exon 10	F419S	c.1256T>C	1	-	Novel
exon 10	I436N	c.1307A>T	2	N	[48]

All sequence information is based on GenBank reference sequence NM_000162.3. Nucleotide numbering reflects cDNA position, with +1 corresponding to the A of the major start codon of exon 1a (present in the pancreatic isoform). Y = yes, N = no.

**Table 2:** Clinical and biochemical parameters of Slovakian *GCK* mutation carriers.

	Probands with *GCK* mutations	Family members with *GCK* mutations	
	*n = 36*	*n = 72*	*Number data available on*
**Age of diabetes diagnosis (yr)**	11.0 (4–45)	26.5 (5–81)	40$
**Age at investigation (yr)**	16.5 (4–47)	35.0 (1–86)	72
**BMI (kg/m^2^)**	19.6 (14.3–29.3)	23.4 (15.2–47.4)	44
**HbA1c (%)**	6.4 (5.1–7.7) *	6.5 (5.0–9.7)	16
**Fasting plasma glucose (mmol/L)**	6.9 (5.5–9.8)	7.0 (5.2–11.8)	65
**Cholesterol (mmol/L)**	4.1 (3.0–5.9)	4.5 (3.0–7.3)	64
**Triglycerides (mmol/L)**	0.8 (0.3–2.4) *	0.9 (0.2–10.0)	64
**HDL-cholesterol (mmol/L)**	1.2 (0.5–2.0)#	1.1 (0.6–2.1)	64
**Treatment before GCK-MODY diagnosis**			42
**-insulin (%)**	19.4	11.9	
**-OHA (%)**	16.7	23.8	
**-diet (%)**	52.8	42.9	
**-none (%)**	11.1	21.4	
**Treatment following GCK-MODY diagnosis**			42
**-insulin (%)**	2.8	2.4	
**-OHA (%)**	5.5	14.3	
**-diet (%)**	80.6	61.9	
**-none (%)**	11.1	21.4	

Data are presented as median values (range).

* Data only available for 35 probands.

# Data only available for 33 probands.

$ For the remaining 32 subjects, diabetes/impaired glucose tolerance was not detected prior to genetic testing.

BMI = body mass index, HbA1c = glycated hemoglobin A1c, HDL = High Density Lipoprotein, OHA = oral hypoglyceamic agents.

### *In silico* analysis of all missense mutations

*In silico* analysis of all missense mutations was performed using a suite of bioinformatic tools. Analysis in SIFT, PolyPhen and Mutation Taster revealed that for the majority of mutations there was consensus between the different bioinformatic tools but for 4 mutations (R43H, V200A, G223S, V244G) at least one program did not predict a clear impact on protein function (**Table S1**). Along with the novel missense mutations these were selected for kinetic evaluation.

### Kinetic Characterisation

The basic kinetic profiles of recombinant wild type and the mutant GCK proteins are given in **table 3**. Seven of the nine mutant proteins (I110N, V200A, N204D, G223S, V244G, G258R, F419S) were shown to be kinetically inactivating, with a decreased rate of catalysis (K_cat_) and/or a decreased affinity for glucose (increased S_0.5_). Of note G258R-GCK and N204D-GKC had extremely low affinities for glucose, with S_0.5_ values of >1000 mmol/L. Interestingly, I110N had an increased affinity for ATP, but was still calculated as being kinetically inactivating as the mutant enzyme had a very low K_cat_. Two mutant enzymes, L315H-GCK and I436N-GCK displayed near-normal kinetics, with similar glucose affinities, Hill number (n_H_), K_cat_, and only a marginally decreased affinity for ATP, compared with the wild-type enzyme.

**Table 3:** Kinetic characterisation of GCK-MODY mutations.

GCK-GST	S_0.5_ [mmol/L]	Hill number	^ATP^Km [mmol/L]	K_cat_ S_0.5_ [s^−1^]	Relative Activity Index (RAI)
WT	7.69±0.10	1.67±0.01	0.44±0.01	65.78±0.84	1
I110N	14.87±0.25	1.37±0.03	0.03±<0.01	3.07±0.04	0.02
V200A**	78.3±0.97	1.28±0.01	0.69±0.07	56.85±0.81	0.01
N204D	NO	NO	5.42±0.10	NO	NO
V244G*	12.77±0.33	1.52±0.02	0.4±0.01	75.71±1.36	0.43
G258R	NO	NO	NO	NO	NO
G223S	16.5±0.16	1.46±0.02	0.47±0.01	74.45±1.63	0.25
L315H	8.09±0.06	1.67±0.01	0.50±0.01	69.21±0.63	0.89
F419S^#^	161.50±1.64	1.12±0.01	6.14±0.23	82.10±0.80	<0.01
I436N	7.84±0.25	1.66±0.02	0.50±0.03	73.77±0.73	1.07

Data are given as mean ±SEM measured in n≥12 experiments. GCK-GST enzymes were prepared as 3 (WT)/2 (G258R)/1 (others) independent protein expressions. Glucose S_0.5_ values were normally measured in the glucose range 0–100 mmol/L with 5 mmol/L ATP, however for some mutant enzymes the glucose range was increased to 0–300 mmol/L (*), 0–600 mmol/L (**), or 0–1000 mmol/L with 25 mmol/L ATP (^#^).

NO = data not obtainable due to the severity of the kinetic inactivation which prevented the data from reaching a plateau even with a 10-fold increase in glucose concentration.

**Figure 1:**
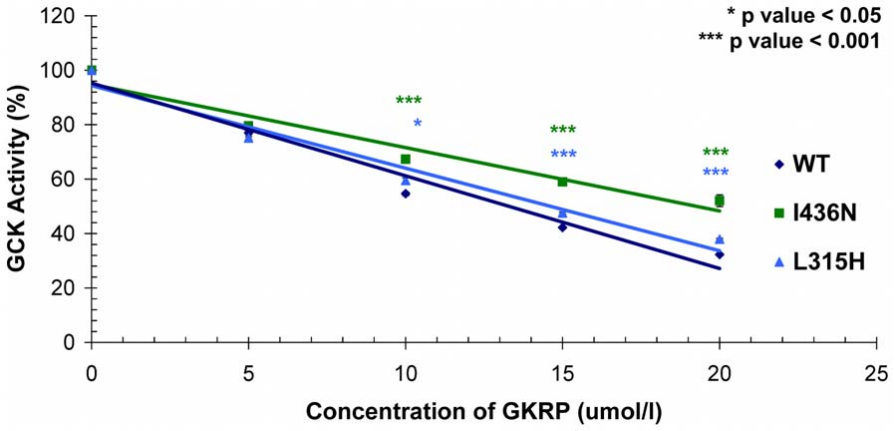
Inhibition of wild type, I436N and L315H glucokinase proteins by human GKRP. Data are shown as mean ±SEM, and were obtained from 4 independent measurements. Independent t-tests were used to ascertain differences between GKRP-mediated inhibition of both mutants versus that obtained with the wild-type GCK enzyme.

To elucidate the molecular mechanism for these two mutants exhibiting near-normal kinetics (L315H and I436N), their interaction with the endogenous hepatic regulator GKRP and their effect on protein stability were explored. Both L315H-GCK and I436N-GCK demonstrated an impaired response to GKRP-mediated inhibition compared with wild-type GCK (**Figure 1**). Accordingly, the IC_50_ values for both mutants were slightly but significantly elevated (14.6±0.1 mmol/L for I436N, 20.3±1.6 mmol/L for L315H, and13.3±0.1 mmol/L for wild-type [both p-values<0.03]).

### Thermal instability assays

**Figure 2:**
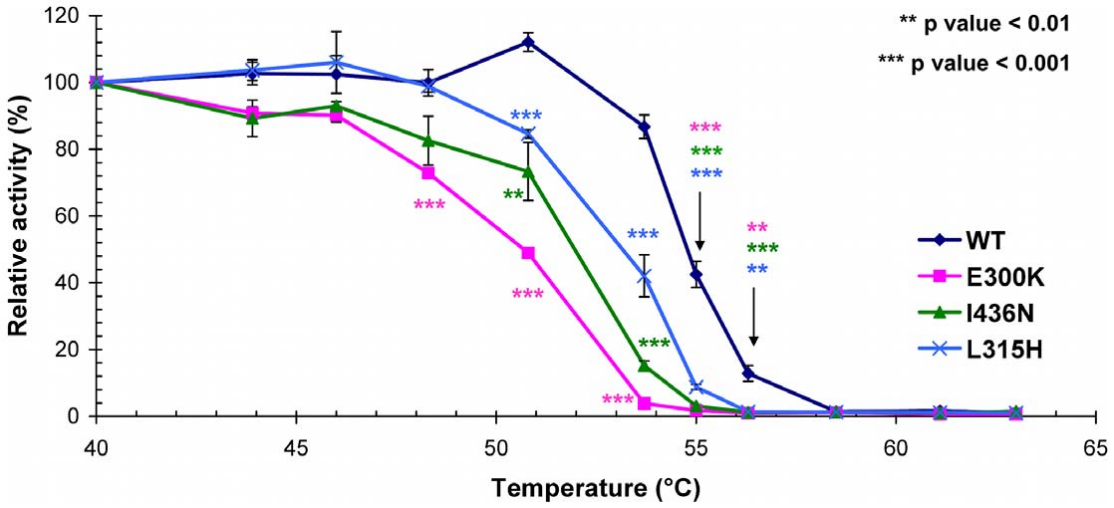
Assessment of thermostability for GCK-GST mutant proteins. Proteins were incubated for 30 minutes over a range of temperatures (40–60°C). Given data are means ±SEM, and were obtained from 6 independent measurements. The previously reported thermal instability mutant E300K [31](pink) was used as a positive control. The level of significance at each temperature point (mutants versus wild-type GCK activity) was calculated using an independent t-test.

To explore whether protein instability could contribute to the mutational mechanism behind the I436N and L315H mutations, thermal-lability assays were performed over a 40–63°C temperature range for both mutant proteins and the known instability mutant E300K-GCK [31]. Analysis of these data showed that both L315H-GCK and I436N-GCK demonstrated marked thermal instability compared to wild-type GCK (**Figure 2**). The relative activity indices of both mutant enzymes was greatly reduced at 55°C, with values of 8.8±0.8% and 3.1±0.4% respectively compared to 42.5±3.9% for wild-type GCK [p<0.001].

### Structural analysis

To understand the extremely low rate of catalysis for N204D-GCK and G258R-GCK we investigated the impact of these mutations on the structure of glucokinase. Modeling established that both residues G258 and N204 are located in close proximity to the glucose binding site. Mutation of residue G258 from glycine to arginine results in obstruction of the glucose binding pocket (**Figure S2**). Structural analysis of the N204 residue revealed that mutation from asparagine to arginine cause a gain of one extra polar bond with residue N254 which is located in the neighboring beta sheet (**Figure S2**).

### Minimum prevalence of GCK-MODY in Slovakia

*GCK* mutations were identified in 36 of the 100 probands screened and mutation testing in family members identified a further 72 individuals with *GCK* mutations. With 337,187 patients with diabetes in Slovakia (Slovak National Health Information Center, 2010), and GCK-MODY confirmed in 108 individuals, this represents 0.03% of all diabetic patients. Recalculated to the number of inhabitants in Slovakia (5.4M, Slovak National Health Information Center, 2008), the minimum prevalence of the GCK-MODY is estimated to be 20.0 cases per million.

## Discussion

It is important to identify patients with GCK-MODY as the diagnosis affects clinical management, prognosis and has implications for family members. We have screened 100 Slovakian probands with a phenotype consistent with GCK-MODY and identified variants in 36% (36/100) of cases. The majority (77%), of variants are missense and a recent study has highlighted the importance of using a combination of family, bioinformatic and functional studies to correctly assign pathogeneticy [19].

Parental DNA was available for 22 of the probands covering 14 of the 22 mutations and co-segregation was established in all cases. In two families there were individuals with diabetes who were not mutation carriers (**Figure S1**). Clinical data supports these individuals as having type 2 diabetes (phenocopies) rather than GCK-MODY (**details given in Figure S1**). A suite of bioinformatic tools (SIFT, PolyPhen, Mutation Taster) were used to assess the impact of all missense variants on protein function. These analyses included conservation of amino acid residues across species (SIFT, Mutation Taster), change in protein structure and function (PolyPhen, Mutation Taster) and effects on splicing or mRNA expression (Mutation Taster) [32], [33], [34]. SIFT and PolyPhen gave similar results predicting the vast majority of mutations to be damaging or possibly damaging (R43H, G223S) and predicting one mutation (V200A) to be tolerated. In contrast the newer program Mutation Taster predicted all mutations to be damaging.

Although bioinformatic prediction softwares are a useful tool the gold standard in assigning pathogenicity to mutations is to demonstrate genetic evidence (i.e. co-segregation in families) and an impact on protein function. Establishing co-segregation for late on-set disorders can be difficult due to problems in correctly assigning affection status and incomplete penetrance. However, with GCK-MODY the assignment of affection status is facilitated by a phenotype (raised fasting plasma glucose) which is easy to measure and presents from birth. Despite this it can still be difficult within a clinical setting to recruit family members for testing. We were able to test parental DNA for 22 of the 36 probands, which covered 14 of the 22 mutations, and confirm co-segregation in all cases. Importantly this included all novel mutations and those with ambiguous *in silico* predictions. To complement our genetic and *in silico* studies we selected all novel missense mutations and those for which there was not a clear consensus on pathogenicity from bioinformatic analysis for further study. The previously reported R43H mutation was not included in our kinetic analysis as recent unpublished studies have shown this mutation to be kinetically inactivating (Nicola Beer, Anna Gloyn unpublished observations) [35].

Basic kinetic characterisation of the mutant glucokinase proteins demonstrated that seven of the nine mutations studied were clearly kinetically inactivating due to either defects in activity or affinity for glucose. Previous studies have also identified *GCK* mutations causal for GCK-MODY which have near normal or paradoxical kinetics [17], [28], [29]. In some cases additional functional studies have demonstrated defects in post-translational regulation by GKRP and small molecular activators and/or protein and catalytic stability [28], [29], [31], [36]. We therefore investigated the effect of the L315H and I436N mutations on the regulation of glucokinase by its regulatory protein GKRP. Our studies demonstrated differences in GKRP inhibition of GCK for both mutations. It has previously been suggested that defects in post-translational regulation of GCK by GKRP can lead to catalytic instability which could contribute to the molecular mechanism for these mutations [36].

Protein instability was assessed using previously validated thermolability assays and demonstrated that both L315H-GCK and I436N-GCK displayed marked thermo-instability which could account for their inactivating properties. Mathematical modeling incorporating this instability predicted that the threshold for GSIR would be ∼7.0 mmol/L for both mutant proteins, which is in line with the clinical data. Further studies in a cellular model would be required to conclusively demonstrate that these mutations results in protein and/or catalytic instability, but our data, supported by previous studies, is highly suggestive of defects in catalytic and protein stability for these two mutants.

For the two proteins N204D-GCK & G258R-GCK for which kinetic parameters could not be fully determined structural modeling gave potential explanations for the extremely low catalytic activity of the two mutant proteins. The glucose binding site is located in the inter domain cleft of the enzyme and is composed of residues of the large domain (E256, E290), the small domain (T168, K165) and connecting region II (N204, D205) [37]. Connecting region II has been proposed to play an important role in the conformational changes required for GCK to move from its inactive to active form [37]. The critical role of residue N204 in the glucokinase molecule is further supported by our studies which show a severe impact of mutation at this residue on enzyme kinetics and additionally by the clinical phenotype of GCK-MODY observed in the patient with the N204D *GCK* mutation. Structural analysis of G258R-GCK, revealed that this mutation also lies in close proximity to the glucose binding site and that mutation from glycine to arginine results in occlusion of the glucose binding pocket and a subsequent spatial barrier for glucose binding.

The minimum prevalence of *GCK* mutation carriers was determined as 0.03% of all diabetic patients in Slovakia and is to date the first estimation of prevalence of GCK-MODY in a diabetic population. The prevalence of *GCK* mutation carriers calculated from an Austrian-German diabetes registry was 0.4% but this study only included patients under the age of 20 years [38]. We calculated the minimum prevalence of the GCK-MODY in Slovakia as 20.0 cases per million which is higher than in two recent studies from the UK [39], [40]. This number is still very likely to be under estimated since a substantial proportion of cases will remain undiagnosed as GCK-MODY is largely an asymptomatic disorder. Shield and colleagues reported 6.4 cases per million with GCK-MODY [39]. The minimum prevalence of GCK-MODY determined in Slovakia (one *GCK* mutation carrier for every 53, 763 inhabitants) is also higher than approximations from Kropff and colleagues who found among one mutation carrier per 118, 927 inhabitants [40]. The median age of diabetes diagnosis in the current study was younger than that of the general diabetic population. Previous work has demonstrated a higher pick up rate for GCK-MODY in patients diagnosed at a younger age [13], [14]. However, since GCK-MODY is often asymptomatic it is likely that we have still underestimated the prevalence of GCK-MODY as our study only includes individuals who have been referred by a Diabetologist for genetic screening. Population studies are required to accurately determine the prevalence of *GCK* mutations. Interestingly, two non-synonymous mutations, L315H and I436N, have previously been identified only in Czech families, and a recent study showed that L315H is one of the most prevalent *GCK* mutations in the Czech population [41]. This finding could suggest a potential existence of founder effect in Slovak and Czech families.

In conclusion, we provide the first evaluation of the prevalence of GCK-MODY in Slovakia and demonstrate that family, bioinformatic and functional studies can be used in combination to assign pathogenicity to missense *GCK* mutations.

## Supporting Information

Figure S1 **Pedigrees demonstrating co-segregation data for GCK mutations with elevated fasting glycaemia for 19/36 probands.** Co-segregation data for 3 families with the −71G>C promoter mutation has previously been reported [42]. Squares represent males, circles females, shaded shapes represent individuals with fasting hyperglycaemia. Fasting plasma glucose levels (mmol/L) are given in blue underneath each individual. Symbols shaded ingrey –represent individuals with fasting hyperglycaemia but with no *GCK* mutation (phenocopies). Probands are indicated by an arrow and the letter P. IFG denotes individuals with impaired fasting glycaemia. NA = not available, NT = not tested, * = not monitored for IFG or DM. Additional clinical information on subjects with fasting hyperglycaemia but no *GCK* mutations: **Family R92 II:3** - lipid profile: cholesterol 8.72 mmol/L (ref. <5.25), triglycerides 9.62 mmol/L (ref. <1.7), HDL-cholesterol 0.82 mmol/L (<1.0). BMI 29.4 kg/m^2^; **Family R227 I:2** - BMI 32 kg/m^2^, age of diagnosis 62 years, currently treated with metformin, also treated for hypertension and dyslipidemia
(TIF)

Figure S2 **Structural analysis of wild-type, G258R and N204D GCK mutants.** **A** The closed (active) crystal structure of glucokinase (1v4s [37]) is shown in green, with bound-glucose represented by black spheres and GKA (yellow) bound in allosteric activator site. **B** Glucokinase structure zoomed to residue 258, where wild-type amino acid glycine and mutant arginine is represented by red and pink respectively. **C** Glucokinase structure zoomed to residue 204, with represented side-chain of all amino acids in polar contact. Wild type amino acid N204 (red) showed polar bonds (yellow) to amino acids V207 (blue) and A208 (orange) and 4 solvent molecules (light blue spheres). Mutagenesis to D204 caused a loss of one of two polar bonds of the solvent molecule and a gain of polar bond (indicated by yellow arrow) with N254 (cyan) which is located in the neighboring beta sheet.
(TIF)

Table S1 **In silico analysis of all missense mutations found in Slovakian patients.**
(DOC)
