# Supplementary figures and images for: Identification and Functional Characterisation of Novel Glucokinase Mutations Causing Maturity-Onset Diabetes of the Young in Slovakia
Source: PLoS One. 2012 Apr 6;7(4):e34541. doi: 10.1371/journal.pone.0034541 (PMC3321013; doi:10.1371/journal.pone.0034541)

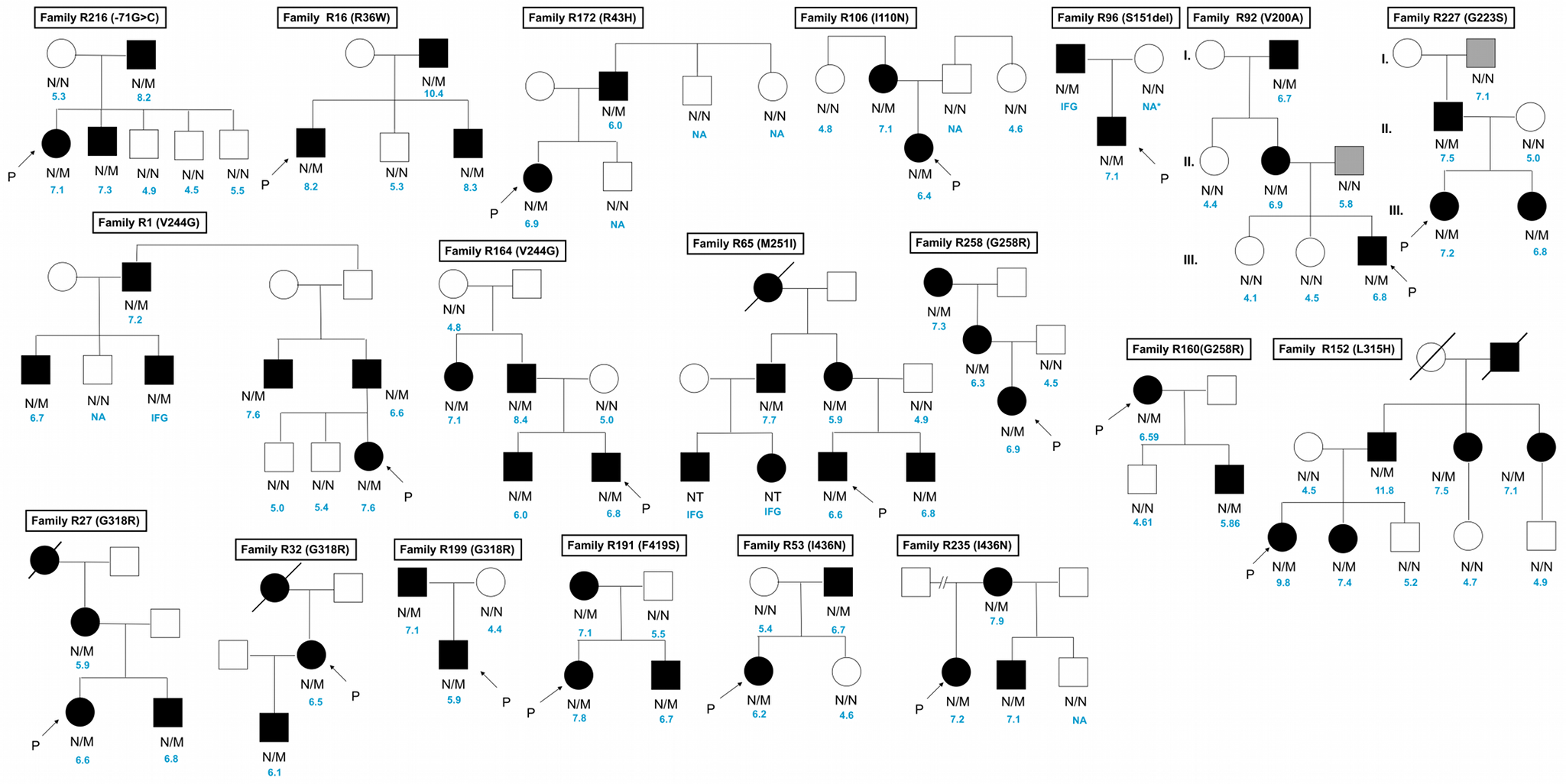

Supplement: Figure S1 — Pedigrees demonstrating co-segregation data for GCK mutations with elevated fasting glycaemia for 19/36 probands. Co-segregation data for 3 families with the −71G>C promoter mutation has previously been reported [42]. Squares represent males, circles females, shaded shapes represent individuals with fasting hyperglycaemia. Fasting plasma glucose levels (mmol/L) are given in blue underneath each individual. Symbols shaded ingrey –represent individuals with fasting hyperglycaemia but with no GCK mutation (phenocopies). Probands are indicated by an arrow and the letter P. IFG denotes individuals with impaired fasting glycaemia. NA = not available, NT = not tested, * = not monitored for IFG or DM. Additional clinical information on subjects with fasting hyperglycaemia but no GCK mutations: Family R92 II:3 - lipid profile: cholesterol 8.72 mmol/L (ref. <5.25), triglycerides 9.62 mmol/L (ref. <1.7), HDL-cholesterol 0.82 mmol/L (<1.0). BMI 29.4 kg/m2; Family R227 I:2 - BMI 32 kg/m2, age of diagnosis 62 years, currently treated with metformin, also treated for hypertension and dyslipidemia (TIF) [file pone.0034541.s001.tif]

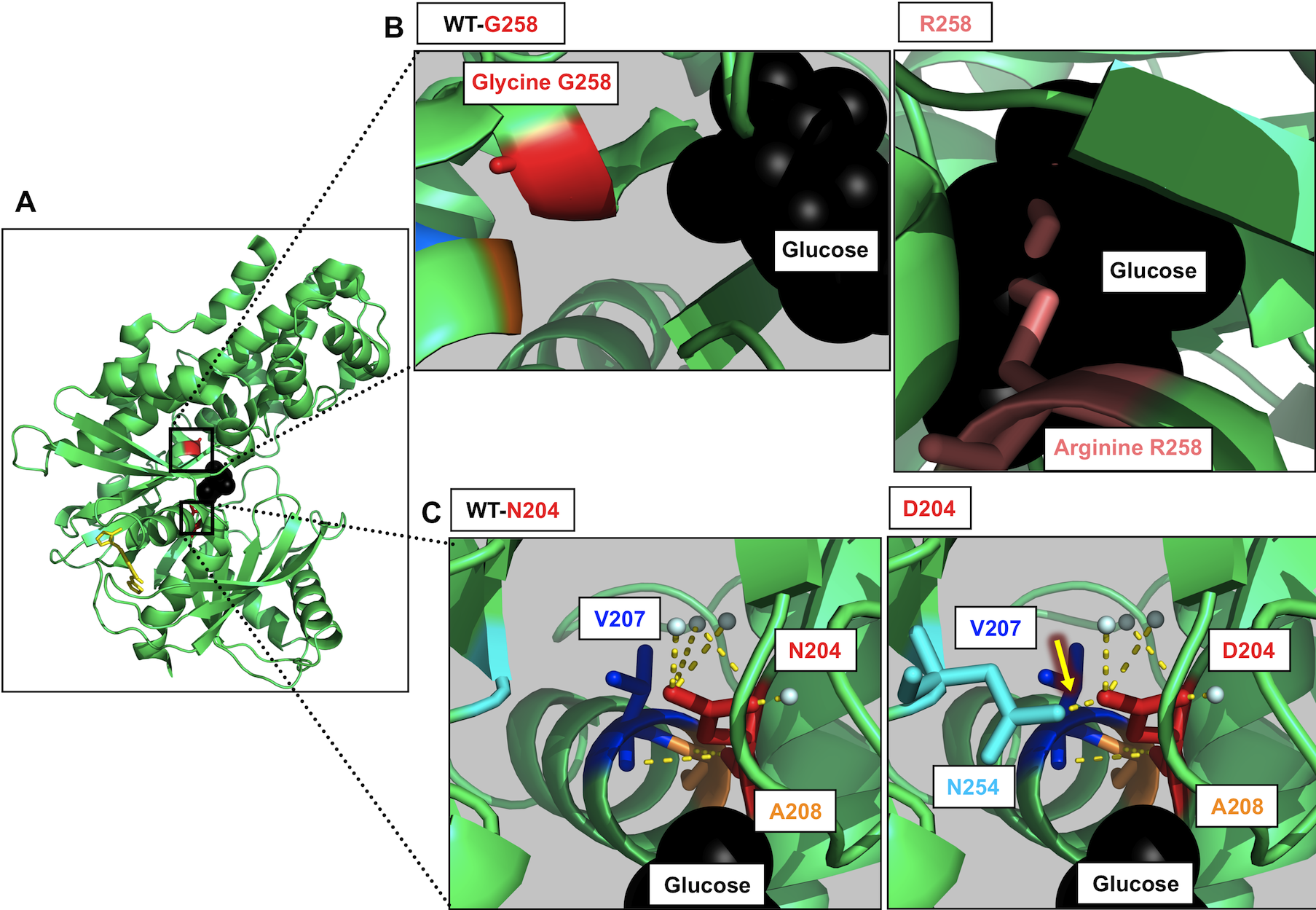

Supplement: Figure S2 — Structural analysis of wild-type, G258R and N204D GCK mutants. A The closed (active) crystal structure of glucokinase (1v4s [37]) is shown in green, with bound-glucose represented by black spheres and GKA (yellow) bound in allosteric activator site. B Glucokinase structure zoomed to residue 258, where wild-type amino acid glycine and mutant arginine is represented by red and pink respectively. C Glucokinase structure zoomed to residue 204, with represented side-chain of all amino acids in polar contact. Wild type amino acid N204 (red) showed polar bonds (yellow) to amino acids V207 (blue) and A208 (orange) and 4 solvent molecules (light blue spheres). Mutagenesis to D204 caused a loss of one of two polar bonds of the solvent molecule and a gain of polar bond (indicated by yellow arrow) with N254 (cyan) which is located in the neighboring beta sheet. (TIF) [file pone.0034541.s002.tif]
