# Supplementary material for: Identification and Functional Characterisation of Novel Glucokinase Mutations Causing Maturity-Onset Diabetes of the Young in Slovakia
Source: PLoS One. 2012 Apr 6;7(4):e34541. doi: 10.1371/journal.pone.0034541 (PMC3321013; doi:10.1371/journal.pone.0034541)
Supplement: Table S1 — In silico analysis of all missense mutations found in Slovakian patients. (DOC) [file pone.0034541.s003.doc]

**SUPPLEMENTARY TABLES**

**Table 1: *In silico* analysis of all missense mutations found in Slovakian patients.**

| **Mutation** | **SIFT** | **PolyPhen** | **Mutation taster** |
| --- | --- | --- | --- |
| R36W | affect function | damaging | disease causing |
| R43H | tolerated | possibly damaging | disease causing |
| G72R | affect function | damaging | disease causing |
| I110N | affect function | damaging | disease causing |
| A188T | affect function | damaging | disease causing |
| V200A | tolerated | tolerated | disease causing |
| N204D | affect function | damaging  disruption of ligand binding site | disease causing |
| T206P | affect function | damaging | disease causing |
| G223S | affect function | possibly damaging | disease causing |
| T228M | affect function | damaging  disruption of ligand binding site | disease causing |
| V244G | affect function | possibly damaging  cavity creation at buried site | disease causing |
| M251I | affect function | damaging | disease causing |
| G258R | affect function | damaging | disease causing |
| L315H | affect function | damaging | disease causing |
| G318R | affect function | damaging | disease causing |
| F419S | affect function | damaging | disease causing |
| I436N | affect function | damaging | disease causing |
